# Book Review

**Published:** 1924-10

**Authors:** 


### Reviews of Books.

---

**Organic Substances, Sera and Vaccines.** By D. W. CARMALT JONES, M.D. (Oxon.), F.R.C.P. (Lond.). Pp. viii., 393. London: William Heinemann. 1924. Price 15s. net.—This book appears to have been designed to meet the want of the general practitioner who desires a small volume which will give advice on the uses of remedies acting in a biological rather than in a chemical sense, both terms being applied loosely. The author deals with endocrine substances, specific and other sera, proteins, vaccines and toxins. The ground to be covered is wide, and much skill was necessary to limit the result to a small single volume. A few points deserve attention. Misprints are rather numerous, especially in the spelling of the names of persons. The opening paragraph to chap. iii. requires revision. Loewi's test as described would be positive in about 90 per cent. of normal subjects; in this test the dilatation of the pupil following the second application of adrenaline constitutes a positive result. The footnote on p. 182 appears obscure. In the rationale of normal serum therapy the explanation given is probably incorrect: a few cubic centimetres of normal serum are hardly likely to contain sufficient coagulative adjuvants to give such dramatic results. The addition of anti-anaphylactic substances, including normal saline, when serum is to be administered intravenously might be stressed. The importance of haste in the transmission of dysentery and other stools to the laboratory requires much more emphasis. On anthrax the author's pessimism regarding the utility of treatment other than specific serotherapy is hardly justified. In his review of vaccine treatment a more optimistic note might have resulted from extracts from more modern articles: thus in the treatment of typhoid fever and pneumonia a good deal of recent work appears to confirm the opinion of the utility of vaccines. Lastly, the index might be enlarged and improved by cross references. These, however, are minor points, and should be remedied in the second edition. On the other scale the author has treated his subjects carefully and cautiously; undue optimism is repressed, a point of some importance in this branch of therapeutics. The section on the collection and transmission of specimens for diagnosis is good, and might be lengthened with profit both to practitioner and pathologist. The work is well up to date; recent gains are noted with caution ; one omission, perhaps intentional, is the phenomenon of Twort-Herelle. The whole book is good. The author has succeeded in his attempt, if the reviewer's opinion of his aim is correct. The work should be of marked practical value.

**Venereal Disease. Its Preventive Symptoms and Treatment.** By WANSEY BAYLEY, M.C. Pp. xvi., 176. London: J. & A. Churchill. 1924. Price 7s. 6d. net.—In this little book the author has placed before his readers an enthusiastic exposition of the important question of venereal disease. It contains a full and fascinating description of the essentials for the prevention, diagnosis and treatment of venereal disease presented to the practitioner in a clear and simple manner. That this edition is the outcome of honest hard work is obvious. Whilst it omits certain tests which are considered unreliable, such as the complement fixation and cultural tests for gonorrhœa, it is most up to date in its reference to such recent work as Professor Levaditi's on bismuth and Professor Fourneau's on stovassol. The methods of prevention advocated are sound and practical.

**Gonorrhœa.** By DAVID THOMSON, O.B.E., M.B., Ch.B. Edin., D.P.H. Camb. Pp. xiv., 519. London: Henry Frowde and Hodder and Stoughton. 1923. Price £2 2s. 0d. net.—This volume is the outcome of seven years' work by the writer on the bacteriology of gonorrhœa. He has given the most complete work that has yet appeared on the subject from any British author. In the main the book is a treatise on the bacteriology of the disease, and more than half of the work is devoted to laboratory methods. His chapters on the cultivation of the gonococcus, its toxins and associates, are excellent. In a later chapter he deals very fully with immunisation by means of vaccine therapy, and favours the use of specific vaccines. Another chapter is devoted to a full account of his work on detoxicated vaccines, and he answers his critics clearly and convincingly. He has sought the assistance of first-class authorities in writing the chapter on vaccine treatment, as well as in those on other forms of modern treatment, including abortive methods. These chapters are eminently practical, and may appeal to a larger circle of practitioners and students than the bacteriological portions of the book. The illustrations, especially the coloured plates, are excellent. The volume provides both attractive and instructive reading, and must have entailed an enormous amount of honest work.

**Notes on the Composition of Scientific Papers.**—By The Right Hon. Sir T. CLIFFORD ALLBUTT. P.C., K.C.B., M.A., M.D., etc. Pp. xii., 192. London: Macmillan & Co. Ltd. 1923. Price 6s.—Those who were already familiar with Sir

Clifford Allbutt's little book of urbane and polished admonitions, in its earlier form, will not be surprised that a new edition has been called for. It is not only that it is alone in its particular field. There is also a charm of style which is in itself the best possible medium for the lesson that its author wishes to teach. Both by example and by precept, however, he sets such a standard of excellence as may well be the despair of lesser mortals, and one might be tempted to remark cynically that not the least merit of the book is that it should serve to discourage persons from the attempt to compose scientific papers. It is an unfortunate fact that many papers are written which ought not to have been written, while many are left unwritten which ought to have been written. We believe that the reading of this book would inspire many to avoid both kinds of error, and for this as well as for other good reasons we commend it to everyone engaged in the struggle against disease.

